# GLP-1 receptor agonist for the management of metabolic syndrome in an adolescent with bipolar disorder on atypical antipsychotic treatment

**DOI:** 10.1192/j.eurpsy.2026.11126

**Published:** 2026-07-31

**Authors:** M. Ruiz Martínez, M. Manjón Núñez, M. Sánchez Picó, M. García Oñoro, S. Rubio Pérez, A. Marcos Merino, C. Del Baño Hernández, E. Aparicio Castro

**Affiliations:** Psychiatry, Hospital General Universitario de Elche, Elche, Spain

## Abstract

**Introduction:**

Metabolic syndrome is a frequent complication in adolescents with bipolar disorder treated with second-generation antipsychotics. Although some agents, such as aripiprazole, have a more favorable cardiometabolic profile, environmental factors, unhealthy eating habits, and behavioral disturbances may contribute to the development of obesity, dyslipidemia, and impaired glucose tolerance. In this context, glucagon-like peptide-1 receptor agonists (GLP-1 RAs) have emerged as a promising therapeutic alternative for weight and metabolic control in pediatric populations.

**Objectives:**

To describe the clinical and metabolic course of an adolescent with bipolar disorder treated with a mood stabilizer and a low cardiometabolic risk atypical antipsychotic, who developed severe treatment-resistant obesity and showed a favorable response to semaglutide.

**Methods:**

We present the case of a 15-year-old male followed by Pediatric Endocrinology and Child & Adolescent Psychiatry. Anthropometric, laboratory, behavioral and therapeutic data were collected over a 7-year period, with particular focus on the evolution after initiation of subcutaneous semaglutide.

**Results:**

The patient was firstly evaluated for obesity at age of 8, with initial improvement under dietary intervention. At age 11, he relapsed with worsening cardiometabolic status (BMI 30.4 kg/m^2^, triglycerides 290 mg/dL, fasting glucose 108 mg/dL) in an adverse family environment and with behavioral difficulties limiting adherence. At age 13, subcutaneous semaglutide was started, leading to weight loss, BMI reduction, and decreased food-related anxiety. Following admission to an intensive therapeutic center, he achieved normal weight (BMI 24 kg/m^2^) and the drug was withdrawn in favor of lifestyle interventions. However, after returning home, obesity recurred (BMI 27.6 kg/m^2^) along with hyperphagia and disruptive behaviors requiring police involvement and psychiatric hospitalizations. Given the previous favorable response, semaglutide was restarted in close coordination with Psychiatry and Pharmacy services. Eventually, custody was transferred to child protection services, and a new specialized residential placement was planned. Throughout the process, the patient remained on therapeutic-range valproate and aripiprazole.

**Conclusions:**

This case highlights the complexity of managing metabolic syndrome in adolescents treated with atypical antipsychotics, even with low cardiometabolic risk agents such as aripiprazole if environmental factors are not suitable. GLP-1 RAs, such as semaglutide, represent an effective and safe strategy for weight control and metabolic improvement in this population. Nevertheless, long-term efficacy requires a comprehensive approach integrating pharmacotherapy with psychoeducation, family involvement, and multidisciplinary coordination between Psychiatry, Endocrinology and Social Services.

**Disclosure of Interest:**

M. Ruiz Martínez Employee of: I declare a direct professional relationship with the patient presented in this work, in my capacity as the treating physician. However, I have not received any funding, nor have I been subjected to any external pressure that could have influenced the preparation, interpretation, or presentation of the results., M. Manjón Núñez Employee of: I have not received any funding, nor have I been subjected to any external pressure that could have influenced the preparation, interpretation, or presentation of the results., M. Sánchez Picó Employee of: I have not received any funding, nor have I been subjected to any external pressure that could have influenced the preparation, interpretation, or presentation of the results., M. García Oñoro Employee of: I have not received any funding, nor have I been subjected to any external pressure that could have influenced the preparation, interpretation, or presentation of the results., S. Rubio Pérez Employee of: I have not received any funding, nor have I been subjected to any external pressure that could have influenced the preparation, interpretation, or presentation of the results., A. Marcos Merino Employee of: I have not received any funding, nor have I been subjected to any external pressure that could have influenced the preparation, interpretation, or presentation of the results., C. Del Baño Hernández Employee of: I have not received any funding, nor have I been subjected to any external pressure that could have influenced the preparation, interpretation, or presentation of the results., E. Aparicio Castro Employee of: I have not received any funding, nor have I been subjected to any external pressure that could have influenced the preparation, interpretation, or presentation of the results.

